# Livestock Development in Hanoi City, Vietnam—Challenges and Policies

**DOI:** 10.3389/fvets.2020.00566

**Published:** 2020-09-10

**Authors:** Long Pham-Thanh, Ulf Magnusson, Minh Can-Xuan, Hung Nguyen-Viet, Åke Lundkvist, Johanna Lindahl

**Affiliations:** ^1^Department of Biosciences, International Livestock Research Institute (ILRI), Hanoi, Vietnam; ^2^Department of Medical Biochemistry and Microbiology, Zoonosis Science Center (ZSC), Uppsala University, Uppsala, Sweden; ^3^Department of Animal Health, Ministry of Agriculture and Rural Development, Hanoi, Vietnam; ^4^Department of Clinical Sciences, Swedish University of Agricultural Sciences, Uppsala, Sweden; ^5^Hanoi Sub-Department of Livestock Production and Animal Health, Hanoi, Vietnam

**Keywords:** urban livestock development, challenges, Hanoi, Vietnam, urban livestock keeping, urban and peri-urban agriculture, food security

## Abstract

The rapid urban growth of Hanoi city requires a livestock production system that ensures both food security and the livelihoods of dwellers. This paper reviews the existing policies for livestock production of Hanoi city and the changes in livestock population between 2014 and 2018 and identifies major challenges for livestock development of the city. While a remarkable increase of the livestock population in recent years is evident, the dominance of small-scale farms, the presence of animal diseases, the slow progress of transiting farms out of urban areas, as well as the lack of analysis of climate change and gender impacts are major challenges that could affect the livestock development of Hanoi.

## Introduction

More than half of the world's population already live in cities, and by 2030, the figure is expected to be 60% (1). Urban migration is partly the result of perceived economic benefits, but there are a number of potential negative impacts. These include an increase in food insecurity and malnutrition, increased health risks, and environmental pollution (2–4). Urban and peri-urban livestock keeping can provide employment and an access to perishable nutritious food that may otherwise be hard to procure, particularly in countries with lacking infrastructure and cold chains. However it can also contribute to the transmission of zoonotic infections and to sanitary problems and conflicts (5–7).

Policies for livestock development may create appropriate incentives for good practice and transparent investment frameworks that take into account all potential benefits and risks. Conversely, if the policies are not well developed and applied, the livestock sector will grow in a non-adequate manner without considering potential risks (8). Many low- and middle-income countries such as Kenya (9), Tanzania (10), Bangladesh (11), India (12), and Vanuatu (13) have made their own national livestock policies, but policies at state or provincial levels are rarely found.

Hanoi, the capital of Vietnam, is in the northern region of the country covering an area of 3,358.6 km^2^ with 7.9 million of permanent inhabitants, including 3.9 million in urban and 4 million in peri-urban areas in 2018 (14). In addition, the city is estimated to receive more than 3 million tourists, travelers, and workers every year. There are 30 districts of the city classified into 12 urban districts and 18 rural/peri-urban districts. Gross regional domestic product (GRDP) of Hanoi in 2018 was about 40 billion USD, of which the livestock sector contributed ~1% (14). Notwithstanding the livestock production growth at about 4.3% per year during 2010 to 2017, it could supply about 60% of the demand of the city burgeoning population (15).

Like other cities in emerging economies, the rapid urbanization of Hanoi requires means to safeguard the urban agriculture, taking into account aspects of productivity, product quality, efficiency, food security, market demand association, income for farmers, and environmental protection (the Resolution number 03/2012/NQ-HDND in April 5, 2012 of Hanoi People's Council).

Hence, the authorities have made a plan for the development of livestock production of the city until 2020 and toward 2030 (the Decision No. 1835/QD-UBND in February 25, 2013 of Hanoi People's Committee). This plan aims to increase the productivity and quality of the animal-sourced food, ensure food hygiene and safety, and create large concentrated commodity production that meets the demand of the consumers and export standards.

This study aimed at understanding the policy vs. the actual situation of the livestock production of Hanoi, as well as to determine potential major challenges of the livestock sector development of the city through policy reviews and key informant interviews.

## Methods

The national livestock development strategy of Vietnam toward 2020 was decided in 2008 (Prime Minister's Decision number 10/2008/QD-TTg). Based on this central direction, some local governments officially issued their own livestock policies (16–18).

Hanoi city provided a strategy for livestock production by 2020 and toward 2030 (19–23). It should be noted that some issues relating to livestock are also regulated in several official documents of Hanoi People's Council (a provincial authority organization) and Hanoi People's Committee (a provincial administrative agency).

**Figure 1:**
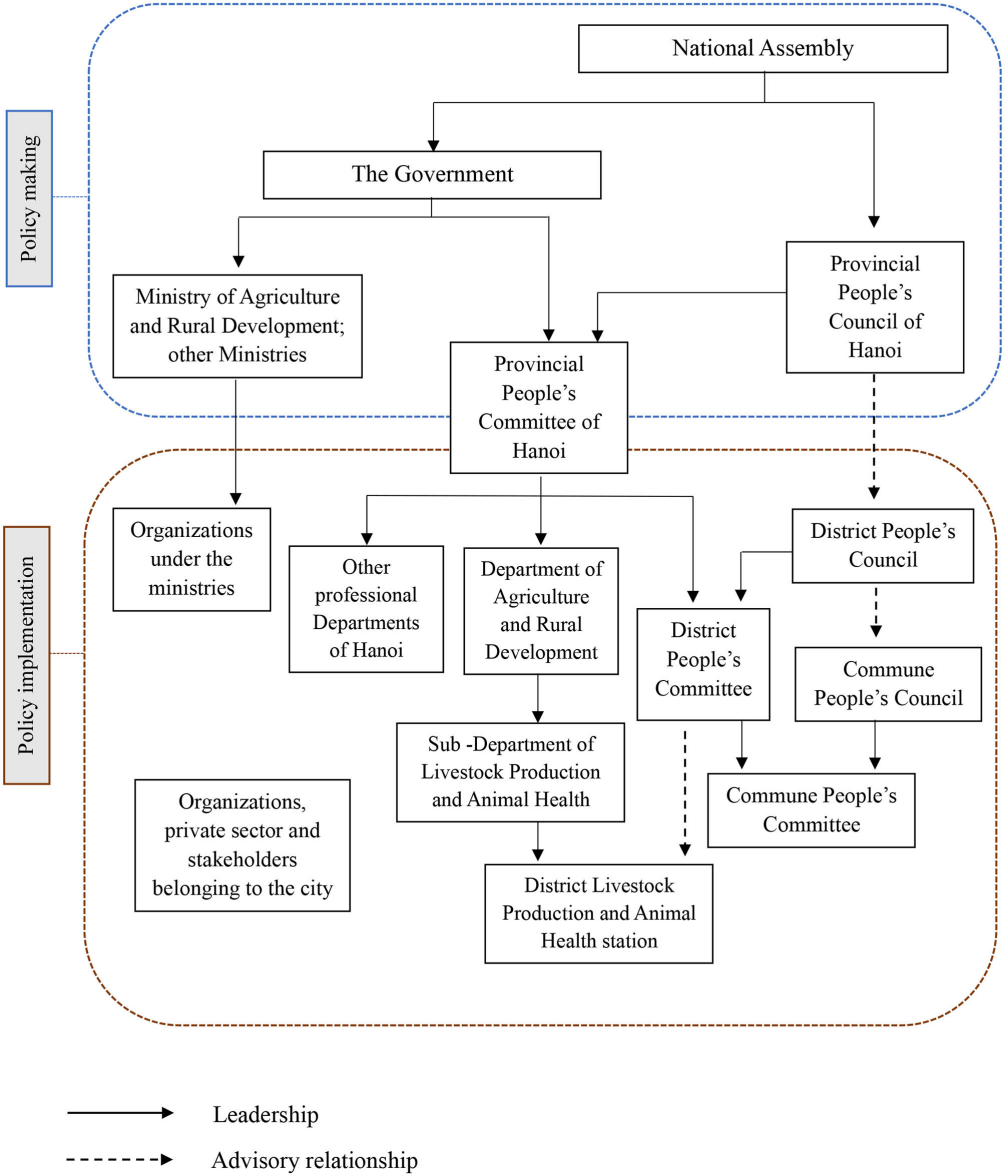
Livestock development policy institutions in Hanoi.

The structure of livestock policy institutions in Hanoi is expressed in Figure 1, and key authorized papers on livestock development of Hanoi are listed in Table 1. In order to conduct a livestock policy analysis of Hanoi, a method combining interviewing key informants from provincial agencies and collecting documented data from them was applied. Six representatives from the Sub-Department of Livestock Production and Animal Health (SDLPAH) under the management of Department of Agriculture and Rural Development (DARD) and chiefs of Livestock Production and Animal Health station from five districts of Hanoi were interviewed via telephone to understand their views on the contents of livestock development strategy, advantages vs. challenges in the current livestock system, zoonotic threats caused by urban livestock keeping, and their suggestions for policies that could maintain livestock sustainability in the future. A recording device and taking written notes were applied during an interview and then transcribed verbatim for analysis. Key answers for current livestock development in these districts were collated in the livestock policy of the city while constraints were grouped with challenges of livestock production of Hanoi.

**Table 1:** The official documents on livestock development of Hanoi issued by local authorities.

**Name of document**	**Organization that authorized**	**Time of issuing**
Resolution number 03/2012/NQ-HDND on the agricultural development plan of Hanoi city till 2020, orientation in 2030	Hanoi people's council	April 5, 2012
Decision number 1835/QD-UBND on approving the plan on development of Hanoi husbandry by 2020, orientation in 2030	Hanoi people's committee	February 25, 2013
Resolution number 10/2018/NQ-HDND regarding some policies to promote production and cooperation development, linkage of production and consumption of agricultural products, building rural infrastructure of Hanoi city	Hanoi people's council	December 5, 2018
Decision number 07/2019/QD-UBND on the issue of mechanism and policy for agricultural production assistance after natural disasters and animal epidemics in Hanoi city	Hanoi people's committee	April 11, 2019
Document number 2933/UBND-KT on management and restructuring livestock husbandry of the city	Hanoi people's committee	July 11, 2019

Legal documents on livestock development of Hanoi People's Council and Hanoi People's Committee were collected, and livestock data were referred from Hanoi Statistics Office at provincial levels and the SDLPAH office for district levels.

Ethical approval for the interviews was obtained from the Ethical Review Board for Biomedical Research of Hanoi School of Public Health (Number 406/2018/YTCC-HD3). The public veterinarians were informed that they could refuse to provide answers to the questions.

## Results and Discussion

### Livestock Production of Hanoi

The general direction in the current policies for the development of livestock is toward industrial farms, which are the large-scale farms applying industrial techniques, concentrated outside the most highly populated areas *(Resolution No.03/2012/NQ-HDND in 2012 of Hanoi People's Council)*. More than 70% of animal products at local markets are expected to be produced by these large-scale farms by 2020. In the short-term, the pig population is planned to be reduced gradually, poultry stabilized, while dairy cow, and beef cattle are to be further developed. The growth rate of animal production is planned to be maintained in the period of 2012 to 2020 at ~1.6% per year and then reduced to 1.4% per year during 2021 to 2030. The livestock sector is planned to contribute to more than 54 and 58% of the agriculture gross output by 2020 and 2030, respectively. Livestock meat supply of the city is expected to be 420,000 tons in 2020 and increases to 492,000 tons in 2030 (*Decision No. 1835/QD-UBND in 2013 of Hanoi People's Committee*). The plan of livestock production in 2020 and in 2030 is shown in Table 2.

**Table 2:** Livestock production trends during 2020 and 2030 in Hanoi.

**Livestock species**	**Year 2020**	**Year 2030**	**Intensify area**
Pigs	1.5 mil. heads; 342,000 tons of pork; <40% are small-scale farms; Supply 8 mil. piglets/year	1.3 mil. head; 340,000 tons of pork; <30% are small-scale farms	Moved to peri-urban area
Beef cattle	155,000 heads; >8,000 tons of beef	From 145,000 to 150,000 heads	Moved to riverbank, hilly areas
Dairy cattle	20,000 heads; 36,000 tons of milk		Rural districts of Ba Vi, Gia Lam, Quoc Oai,Dong Anh, Dan Phuong, and Phuc Tho
Buffaloes	16,000 heads		
Poultry	15 mil. birds; 66,000 tons 80% are chickens; 800 mil. eggs	14.3 mil. birds	Rural districts of Chuong My, Dong Anh, Ba Vi, Quoc Oai, Soc Son, Thanh Oai, Ung Hoa, Phuc Tho, Phu Xuyen, My Duc, and Thach That

The livestock strategy of five districts where the interviews were conducted showed a consistency with the plan of livestock development of the city. In particular, Thach That district is planned for large ruminants and pigs, Phuc Tho district is for cattle and chickens, Ba Vi district is projected for dairy cattle concentration, My Duc district is for waterfowls and beef cattle, and Son Tay district is planned for large-scale pig production by 2020.

#### Land Use for Livestock Production

Productive land is the most fundamental resource required for livestock keeping (24), but in urbanized areas in many developing countries, the limited space for domestic animals is an obstacle (25, 26). According to the United Nations Convention to Combat Desertification (UNCCD), the estimates of prime agricultural land lost to urbanization around the world range from 1.6 to 3.3 million hectares per year in the period between 2000 and 2030.

**Figure 2:**
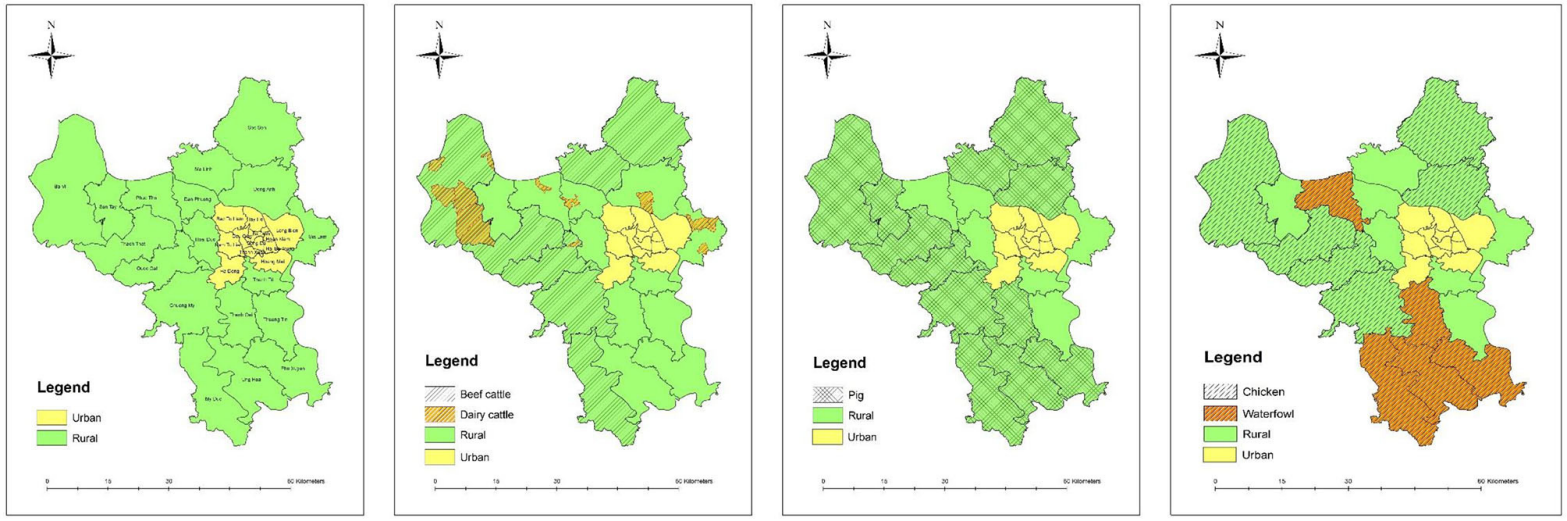
Planned areas for livestock development in Hanoi by 2020.

The spatial distribution of livestock production of Hanoi is planned based on the geography of the rural districts. In brief, the keeping of beef cow, dairy cattle, and fattening pigs is to be concentrated in hilly districts while chickens and waterfowls are to be expanded in plain areas (Figure 2). Translocation of most livestock farms out of residential areas in rural districts as well as removal from urban districts is expected to be achieved by 2030 (*Decision No. 1835/QD-UBND in 2013 of Hanoi People's Committee*).

Due to the rapid urbanization of Hanoi, the area of agricultural land is predicted to be reduced by ~19% from 2014 to 2020 (27).

**Figure 3:**
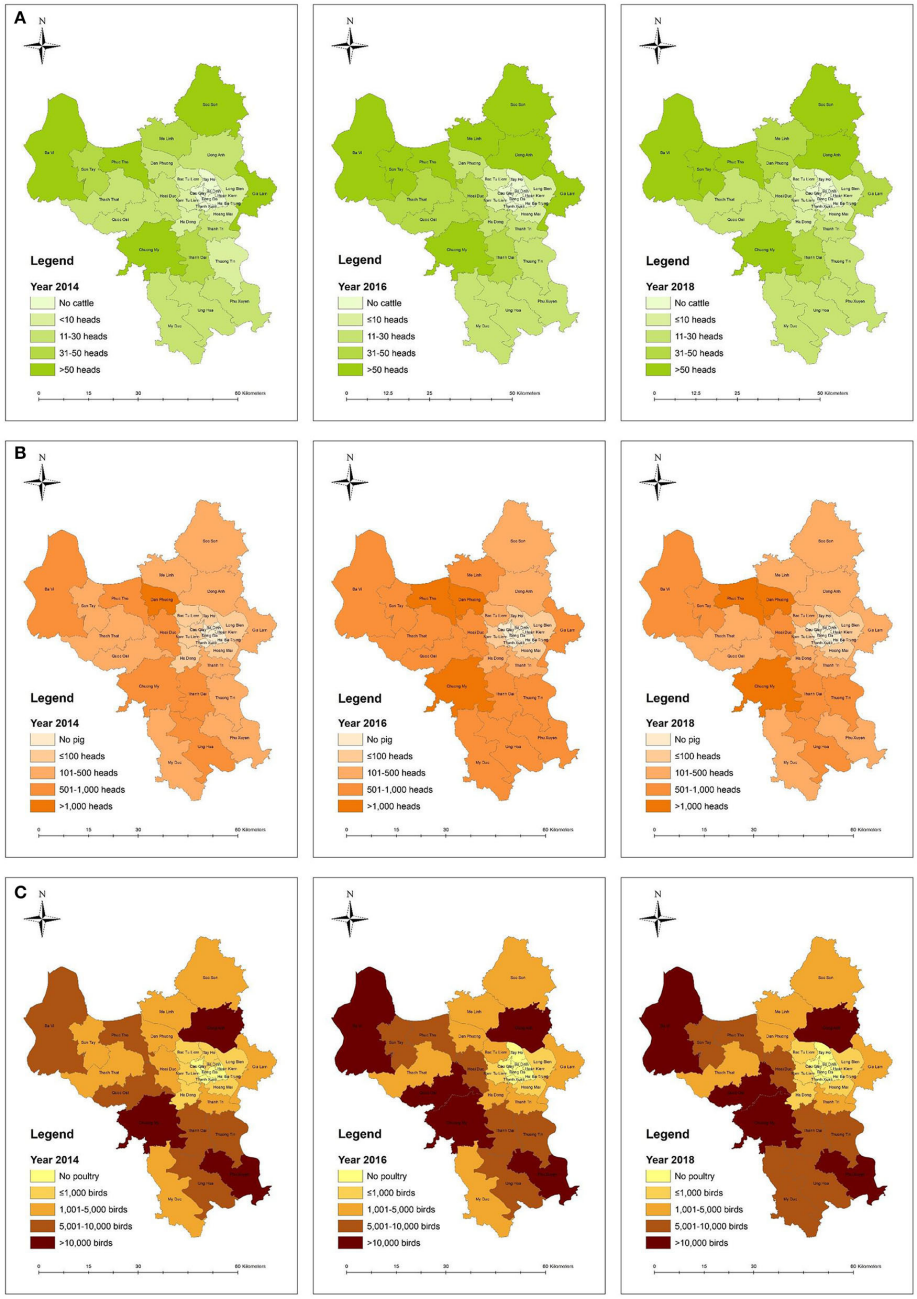
**(A)** Cattle density (head per km^2^) in 2014, 2016, and 2018 in Hanoi (Data source: Hanoi SDLPAH). **(B)** Pig density (head per km^2^) in 2014, 2016, and 2018 in Hanoi (Data source: Hanoi SDLPAH). **(C)** Poultry (head per km^2^) in 2014, 2016, and 2018 in Hanoi (Data source: Hanoi SDLPAH).

Stipulated in the livestock production law of Vietnam, livestock keeping is not allowed in urban and residential areas (28). However, livestock was still present in at least 6 out of the 12 urban districts of Hanoi, as of 2018 (Figures 3A–C). Thus, the goal of moving all livestock farms out of urban areas is not yet fulfilled.

Land ownership is a major barrier affecting livestock translocation from residential areas. The current regulation does not permit a longer rental time of agricultural production land than maximum 5 years (29). This is a very short time period to motivate the private sector to invest in large-scale livestock establishments.

#### Livestock Population

**Figure 4:**
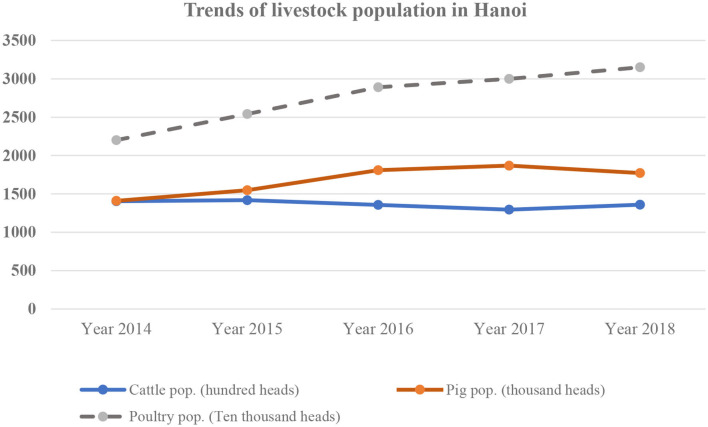
Livestock population in Hanoi during 2014–2018 (Data source: Hanoi Statistics Office).

In 2018, there were 1.8 million pigs, 136,000 cattle, 23,500 buffaloes, 21.8 million chickens, and 6.2 million waterfowls in Hanoi city (14) (Figure 4). The livestock population of the city is planned in the Decision No. 1835/QD-UBND in 2013 of Hanoi People's Committee. By 2020, the beef cattle population is projected to be between 150,000 and 155,000 heads, while dairy cattle should reach 20,000 heads. A stabilized cattle population with 145,000 to 150,000 heads is expected by 2030. The total number of pigs is planned to reach 1.4 to 1.5 million by 2020. Until 2030, the pig population is projected to decrease to 1.3 million, but the pork volume is maintained at 340,000 tons per year, reflecting an expected higher productivity. The total population of poultry is expected to be over 11.6 million chickens and 2.8 million waterfowls providing more than 66,000 tons of meat and 800 million eggs per year by 2020. In 2030, the poultry population is projected to be 14.3 million.

Compared to the expected livestock populations in 2020, pigs and poultry in 2018 were much higher, but in contrast, the cattle production was lower. Livestock population trends from 2014 to 2018 are shown in Figure 4. According to Hanoi SDLPAH, the reduction rate of small-scale farms from 2014 to 2018 in urban areas was higher, 32%, as compared to rural areas, 24%. The changes in number of small-scale farms from 2014 to 2018 are shown in Table 3.

**Table 3:** The number of small-scale farms for livestock keeping during 2014–2018 (Data source: Hanoi SDLPAH).

**Area**	**Year 2014**	**Year 2015**	**Year 2016**	**Year 2017**	**Year 2018**
Urban	4,229	4,096	4,155	3,426	2,871
Rural	396,202	364,283	351,557	331,552	300,879
Total	400,431	368,379	355,712	334,978	303,750

In 2018, there were five large-scale cattle farms in the Hanoi area, accounting for 12% of the total cattle population, 283 large-scale pig farms, accounting for 22% of the total pig population, and 290 poultry farms that kept at least 2,000 birds, which accounted for 8% of the total poultry population. These figures show that the livestock production of Hanoi is facing a big challenge due to the scattered and numerous small-scale farms. The most constraints to livestock development of Hanoi identified by the key informants are small-scale farms. Compared to large-scale farms, small-scale farms have poorer farm biosecurity, higher risk of disease exposure, lower productivity, lower quality of waste treatment, worse quality feed sources and breeds, lower technology investment in livestock production, and more difficult access to the capital (30, 31).

Nevertheless, given the assets and aspirations of farmers, policies toward small-scale farms are still needed (32). Shifting extensive production to semi-intensive and/or intensive production systems is a suggested solution (31).

#### Breeding

According to the Decision No. 1835/QD-UBND in 2013 of Hanoi People's Committee, improvement of cattle, pig, and poultry breeds is a major focus. For breeding of cattle, the programs on artificial insemination, exotic cattle breed import and a breeding program on hybrids between exotic zebu bulls and indigenous cows are planned.

Similar to cattle, the plan for swine breed improvement strongly relies on import of high-quality exotic breeds and artificial insemination program. Investment of pig breeds from the private sector is encouraged additionally. Apart from breeding of purebred sows or crossbred sows for grandparental herds, the production of crossbred for commercial litters is also projected to be enhanced. Moreover, management of semen sources used in private breeder farms and imported semen quality is expected to be strictly controlled by the local authorities.

For poultry breeding, the investment in grandparental flocks at the scale of 5,000 to 10,000 birds per farm to supply hatcheries and the introduction of new high-yielding and high-quality poultry breeders are projected until 2030. The conservation, preservation, and development of native breeds are also planned.

As of 2018, cattle breeding was diverse, with 65% of zebu crossbred cows; about 30% of exotic crossbred cows such as Blanc Blue Belle, Angus, Wagyu, Brahman, Charolais, and Droughtmaster; and 5% of indigenous breed. Artificial insemination of beef cattle covered more than 80% of the services, and produced about 60,000 calves per year (33).

In 2019, exotic sows accounted for 86% of the sow population, and these high-quality sows, imported from France and Denmark, produced ~250,000 piglets per year. The poultry sub-sector delivered about 70 million day-old chicks and 10 million day-old ducks per year (reported by Hanoi SDLPAH in 2019).

There were 340 hatchery establishments for chickens and waterfowls consisting of 224 small-scale and 116 large-scale commercial hatcheries in the city, as of 2018. These hatcheries not only certainly ensure the supply of poultry quantity of Hanoi but also export day-old birds to other provinces.

The quality improvement of livestock breeders of the city faced a challenge because of the high proportion of the small-scale livestock production system. Livestock of small-scale farms strongly depends on the availability of breeder producers and the sustainability of breeder quality and productivity. However, these criteria were not systematically assessed by local authorities. This is in accordance with previous observations stating that many breeding programs in tropical countries have failed when applied to small-scale farming systems (34).

### Animal Feed

In the Decision No. 1835/QD-UBND in 2013 of Hanoi People's Committee, increased production of cattle forages, starch, and supplementary feedstuff is planned. In particular, eight large clusters for forages for dairy cows and beef cattle are projected to be under construction in 10 rural/peri-urban districts. The supply of feed mills in the city is planned for local demand only.

Based on a report of Hanoi SDLPAH, the total number of companies and enterprises producing and trading animal feed in 2018 was 140, in which 96 establishments were located in Hanoi, whereas 44 establishments were registered for business there, but the production was in other provinces. These feed establishments produce sufficiently animal feed to the farmers of the city.

### Veterinary Services in Hanoi

The veterinary services play a key role in livestock production to ensure a high animal health status and food safety (35). However, some constraints caused by animal health services such as shortage of manpower, long distances from service centers, and high costs for services were identified (36).

#### Veterinary System

In the plan of the city, there are investments in both the veterinary service infrastructure and in human resources. In particular, the working office of Hanoi SDLPAH and a veterinary diagnostic laboratory are to be upgraded. In addition, the development of human resources through veterinarian recruitment, professional training, and research is also included in the plan. Animal disease monitoring and animal health information systems are projected to be consolidated through a veterinary network between different administrative levels. Capacity building is needed in data analysis, forecast, disease warning, and diagnostics. Moreover, disease control strategies and disease-free compartmentalization programs are to be implemented (*Decision No. 1835/QD-UBND in 2013 of Hanoi People's Committee*).

Before October 2018, the veterinary service system of Hanoi was managed by the Sub-Department of Animal Health that operated from a village level up to a provincial level through an animal health worker network of more than 3,000 persons. In October 23, 2018, the People's Committee of Hanoi decided to merge the veterinary service system and livestock production agency (the Decision number 5682/QD-UBND on Establishment of Hanoi Sub-Department of Livestock Production and Animal Health). Hence, the number of veterinarians in Hanoi has been reduced at all levels, especially the community-based animal health worker forces that were mostly affected. In some low- and middle-income countries, the community-based animal health workers play an important role in early detection and reporting disease outbreaks, vaccination campaigns against the most important livestock diseases, and providing treatment to sick animals (37, 38).

#### Compensation

According to the policy of the city, the owner of a domestic animal compulsorily culled during a disease outbreak in Hanoi is planned to be compensated with the amount of 1.6 USD per kg of pig; 1.9 USD per kg of cattle, buffalo, goat, sheep, and deer species; and 1.5 USD per bird of chicken, duck, Muscovy duck, and goose species *(Decision No. 07/QD-UBND in 2019 of Hanoi People's Committee)*.

Reported by Hanoi SDLPAH, the city compensated more than 60 million USD to pig farmers whose animals were culled due to African swine fever epidemic in 2019.

#### Other Aspects of Veterinary Services

In the Decision No. 1835/QD-UBND in 2013 of Hanoi People's Committee, apart from consolidation of quarantine stations and checkpoint systems, the aspects relating to animal movement, slaughter inspection, veterinary hygiene, and waste treatment at slaughterhouses, and animal product residues control are planned to be strengthened. Regulations on the production, trading, and use of antibiotics and growth promoters are to be strictly enforced by inspections in parallel with instructions for the farmers on proper use of veterinary medicines. Besides, the dissemination and education on legislations and knowledge on veterinary medicines through the mass media are projected to be performed. In addition, training courses on animal health management, food hygiene, and safety from farms to slaughterhouses are planned to be organized accordingly.

Due to ongoing restructuring of the organization of veterinary service system of Hanoi, these implementations were not assessed in this study.

### Waste Treatment

On one hand, livestock manure is useful for crop production, but on the other hand, livestock waste may be harmful for the environment, and it can also pose a risk for public health (39). There are both technological and natural options for treatment of livestock effluents and manures, but such options need to be adapted and implemented according to the local situation and contexts of society, economy, and regulations (40).

The plan for 2020 in the Decision No. 1835/QD-UBND in 2013 of Hanoi People's Committee includes application of biotechnology, environmental treatment technology in livestock production, and improvement of waste treatment and biogas programs for livestock farm through investment programs.

According to the interviewees, livestock owners in Hanoi are aware of negative impacts of livestock waste such as gas emissions, bad odors and environment pollutions; therefore, they have applied several waste treatment systems in their farms. Manure composting is the most common method followed by biogas system and probiotic applications. The number of farms using livestock waste treatment system of Hanoi in 2018 is shown in Table 4.

**Table 4:** The number of livestock farms applying waste treatment system in 2018 (Data source: Hanoi Department of Agriculture and Rural Development – Hanoi DARD).

Type of waste treatment^*^	**Cattle farm**	**Pig farm**	**Poultry farm**
Biological padding	157	674	11,194
Biogas technology	5,035	103,318	1,681
Manure composting	17,368	12,436	94,678
Probiotics application	2,694	19,735	15,491
Others	1,227	3,543	5,087

* *One farm can apply more than one treatment system*.

### Investment Capital and Financial Support

According to the policy, the total investment during 2012 to 2020 is about 510 million USD with 12% from the government budget, and the remaining 88% from the private sector (Decision No. 1835/QD-UBND in 2013 of Hanoi People's Committee). Besides, the investments from foreign enterprises for livestock development are encouraged.

In particular, investments in grandparental farms for pigs and boar semen establishments, egg processing plants, meat storage bases, waste treatment system, large-scale abattoirs, and semi-line slaughter facilities are projected with a valuable support of interest rate and loan from the city. The government budget is allocated to artificial insemination programs for high-yielding pig and cattle breeds, vaccinations against foot and mouth disease, classical swine fever, porcine reproductive and respiratory syndrome, avian influenza, and disinfectants used in the disease outbreak containment (Resolution No.10/2018/NQ-HDND in 2018 of Hanoi People's Committee).

The data for investment capital and financial support of the plan are not available; therefore, this aspect was not analyzed in this study.

### Other Concerns to Livestock Development of Hanoi

#### Animal Disease

Another major challenge is diseases in the livestock. In 2018, foot and mouth disease, highly pathogenic avian influenza, porcine reproductive and respiratory syndrome, and other animal infectious diseases were reported by Hanoi SDLPAH. According to the data of the Department of Animal Health, during February to December 2019, an epizootic of African swine fever occurred in Hanoi and caused culling of more than 540 thousand pigs, which accounted for more than 25% of the pig population of the city.

Several economic studies have demonstrated huge costs of livestock epidemics such as 9.2 million USD for African swine fever in Cote d'Ivoire in 1996; 114 million USD for Nipah virus in Malaysia from 1998 to 1999; and 300 million USD for contagious bovine pleuropneumonia in Botswana in 1995 (41). Also in Vietnam, a study on avian influenza revealed financial losses ranging from 70 to 108 USD per farm (42). Annual loss by diseases in livestock population has not yet been systematically assessed in Hanoi.

Risk of zoonotic transmission associated with livestock keeping in Hanoi was mentioned during the interviews. The participants listed highly pathogenic avian influenza and *Streptococcus suis* type 2 as the potential zoonoses of the city. Many other zoonotic diseases can occur with urban livestock keeping, including vector borne and food borne, but were not mentioned, potentially reflecting which diseases have been more frequently mentioned in the media.

#### Climate Change

The climate change can affect livestock production through its negative impacts to feed crop quality, water resource availability, and livestock health situation (43). An increase in the number of outbreaks of transboundary animal diseases by climate change has caused loss of several billion USD over the past decades and low-income countries are most affected (44). In addition, Vietnam was on the list of the five most affected countries in the world by extreme weather events in 2016 (45).

There are studies on climate conditions and climate change scenarios for Vietnam, including effects on livestock production, proposed solutions for minimizing losses, and optimizing investment efficiency (46). However, such overall mapping of climate change challenges specific to Hanoi has not yet been conducted.

#### Gender Issue

Both males and females are involved in the small-scale livestock sector. Men usually keep cattle and buffalo, while women are responsible for poultry and small ruminants. However, it is harder for women to access resources, rights, and services for livestock production because of gender discrimination in many low- and middle-income countries (47). At all stages of the urban livestock production, as well as in any development programs, gender concerns need to be addressed (48).

In Vietnam, the gender equality law was approved by the National Assembly in 2006, but there are at present very few studies on gender impact, especially the role of women, in livestock keeping in urban cities, and Hanoi is not an exception.

### Limitations of the Study

The study has some limitations. The data used were from the government agencies and did not include the opinions of urban inhabitants and farmers. Some parameters such as investment capital, disease compensation, technology application in livestock production, and challenges affecting the sustainability of livestock development of Hanoi were not measured due to lack of information.

### Implications

This review about the present situation of urban livestock keeping in Hanoi and the relevant policies may be helpful for other growing cities in need of analyses of their own situation and their policies. In addition, this paper presents data on the importance of urban livestock keeping and the implications of policies on its development, which may be critical for planning of interventions for livestock health and productivity, as well as for food and feed security and safety, and public health aspects.

## Conclusions

During 2014 to 2018, the livestock production system of Hanoi urban city has significantly increased in line with the policy from the authorities. However, some major challenges were identified by this review. The high proportion of small-scale farms of livestock keeping in Hanoi is the biggest constraint for the development of livestock production because it makes several negative impacts including poor animal health and environment management, weak animal breeding improvement, low technological applications, limited access to land or capital resources, and low profits for livestock owners. Unpredictable diseases of domestic animals with the case of an African swine fever epizootic that occurred in Hanoi in 2019 demonstrated that the livestock sector is very vulnerable to contagious diseases. The progress of translocation of livestock farms out of the urban areas is slow, mainly due to the rapid urban growth narrowing agriculture land, current land rental regulations, and investment limitations. The evaluation of the impact of climate change and gender equality is still missing in the livestock policy of Hanoi. An annual livestock policy analysis in Hanoi is highly recommended. The implications for zoonotic disease burden associated with livestock production in urban areas and impacts of animal epidemic outbreaks to livelihood of livestock owners can provide useful information to inform livestock policy of cities in emerging economies.

## Author Contributions

All authors contributed in the conception and design of this study. LP-T did the first draft and all authors reviewed and contributed. JL supervised the project.

## Conflict of Interest

The authors declare that the research was conducted in the absence of any commercial or financial relationships that could be construed as a potential conflict of interest.

## References

[1] United Nations Population 2030: Demographic Challenges and Opportunities for Sustainable Development Planning. United Nations. (2015). Retrieved from: http://www.un.org/en/development/desa/population/publications/pdf/trends/Population2030.pdf (accessed February 28, 2020).

[2] Armar-Klemesu M Thematic Paper 4 Urban Agriculture and Food Security, Nutrition and Health. 99–117. (2015). Retrieved from: http://futuredirections. org.au/wp-content/uploads/2015/05/1391511018Urban_agriculture_adn_ food_security,_nutrition_and_health.PDF (accessed August 2, 2019).

[3] De Bon H Parrot L Moustier P Sustainable urban agriculture in developing countries. A review. Agron Sustain Dev. (2010) 30:21–32. 10.1051/agro:2008062

[4] Hassell JM Begon M Ward MJ Fèvre EM. Urbanization and disease emergence: dynamics at the wildlife–livestock–human interface. Trends Ecol Evol. (2017) 32:55–67. 10.1016/j.tree.2016.09.012 28029378 PMC5214842

[5] Grace D Lindahl J Correa M Kakkar M Urban livestock keeping. In: de Zeeuw H, Drechsel P, editors. Cities and Agriculture: Developing Resilient Urban Food Systems. New York, NY: Routledge (2015). p. 255–84.

[6] Lindahl JF Magnusson U Grace D Urban livestock-keeping: contributions to food and nutrition security. In: Ferranti P, Berry EM, Anderson JR, editors. Reference Module in Food Science. Amsterdam: Elsevier Ltd (2018). 10.1016/B978-0-08-100596-5.21531-X

[7] Lindahl JF Magnusson U Grace D Urban livestock keeping: leveraging for food and nutrition security. In: Ferranti P, Berry EM, Anderson JR, editors. Encyclopedia of Food Security and Sustainability. Amsterdam: Elsevier Ltd (2018). 10.1016/B978-0-08-100596-5.21548-5

[8] Nabarro D Wannous C. The potential contribution of livestock to food and nutrition security: the application of the One Health approach in livestock policy and practice. Rev Sci Tech Off Int Epiz. (2014) 33:475–85. 10.20506/rst.33.2.2292 25707178

[9] KMoLD Ministry of Livestock Development, Kenya. National livestock policy. Nairobi (2008).

[10] TMoLD Ministry of Livestock Development of Tanzania. National Livestock Policy. Dar es Salaam (2006).

[11] MoFL Ministry of Fisheries and Livestock, Bangladesh. National Livestock Development Policy. (2007).

[12] MoA. Ministry of Agriculture, India. National Livestock Policy. (2013).27906123

[13] MALFFB Ministry of Agriculture, Livestock, Forestry, Fisheries and Biosecurity, Vanuatu. National Livestock Policy 2015–2030. (2013).

[14] Hanoi Statistics Office Hanoi Statistical Yearbook 2018. (2019).

[15] Hanoi DARD DARD report. (2019).

[16] Decision No 1350/QD-UBND. Hung Yen province People's Committee. (2012).

[17] Decision No 3178/QD-UBND. Ho Chi Minh city People's Committee. (2011).

[18] Decision No 32/QD-UBND. Quang Binh province People's Committee. (2008).

[19] Resolution No03/2012/NQ-HDND. Hanoi People's Council. (2012). p. 1–7

[20] Decision No 1835/QD-UBND. Hanoi People's Committee. (2013).

[21] Resolution No Q-HDND. Hanoi People's Council (2018).

[22] Decision No 07/QD-UBND. Hanoi: Hanoi People's Committee. (2019).

[23] Document No D-KT. Hanoi People's Committee (2019).

[24] IFAD Improving access to land and tenure security. Palombi e Lanci, Rome, 44. (2008).

[25] Bren d'Amour C Reitsma F Baiocchi G Barthel S Güneralp B Erb KH. Future urban land expansion and implications for global croplands. Proc Natl Acad Sci USA. (2017) 114:8939–44. 10.1073/pnas.1606036114 28028219 PMC5576776

[26] Roessler R Mpouam SE Muchemwa T Schlecht E Emerging development pathways of urban livestock production in rapidly growing West Africa cities. Sustainability. (2016) 8:1199 10.3390/su8111199

[27] Department of Science and Technology Project code 01C-05 on Orientation and Solution for Agricultural Suburban Restructuring in Hanoi in the Period to 2020, vision to 2030. (2016). Retrieved from: https://vanban.hanoi.gov.vn/detaikhoahoc?p_p_id=VsubjectView_WAR_Vsubjectportlet_INSTANCE_1TyasZEx52tW&p_p_lifecycle=0&p_p_state=normal&p_p_mode=view&p_p_col_id=column-1&p_p_col_count=1&_VsubjectView_WAR_Vsubjectportlet_INSTANCE_1TyasZEx52tW_jspPage=%2Fht (accessed August 4, 2019).

[28] National Assembly of Vietnam The Livestock Production Law of Vietnam. Hanoi (2018).

[29] National Assembly of Vietnam The Land Law of Vietnam. Hanoi (2013). p. 63

[30] Baltenweck I Thinh NT Nga NTD Hung PV Nhuan NH Huyen NTT Assessing competitiveness of smallholder pig farming in the changing landscape of Northwest Vietnam. ILRI Research Report. (2018) 52 Available online at: https://hdl.handle.net/10568/98904

[31] Dung DV Roubík H Ngoan LD Phung LD Ba NX Characterization of Smallholder Beef Cattle production system in central vietnam -revealing performance, trends, constraints, and future development. Tropical Ani Sci J. (2019) 42:253–60. 10.5398/tasj.2019.42.3.253

[32] Hazell P Comparative Study of Trends in Urbanization and Changes in Farm Size in Africa and Asia : Implications for Agricultural A Foresight Study of the Independent Science and Partnership Council. Independent Science and Partnership *Council* (2013) 9.

[33] Department of Agriculture and Rural Development Potential for Ruminant Livestock Development in General, Beef Cattle Raising in Particular in Hanoi city. Hanoi: Department of Agriculture and Rural Development (2019).

[34] Kaasschieter GA de Jong R Schiere JB Zwart D. Towards a sustainable livestock production in developing countries and the importance of animal health strategy therein. Vet Quarterly. (1992) 14:66–75. 10.1080/01652176.1992.9694333 1502778

[35] Kwaghe AV Vakuru CT Dika Ndahi M Usman JG Abubakar A Iwar VN Veterinary services as a panacea for agricultural development and increase in nigeria's gross domestic product (GDP): a review. Int J Life Sci. (2015) 4:134–46.

[36] Kebede H Melaku A Kebede E. Constraints in animal health service delivery and sustainable improvement alternatives in North Gondar, Ethiopia. Onderstepoort J Vet Res. (2014) 81:1–10. 10.4102/ojvr.v81i1.713 25685946

[37] Hassan A Restructuring of veterinary services through consolidation of private veterinary practice and introduction of new approaches for intergration of target groups in the Middle East. Conference of OIE. (2001) 5–14.

[38] Holden S. The economics of the delivery of veterinary services. Rev Sci Tech. (1999) 18:425–39. 10.20506/rst.18.2.1166 10472677

[39] Ström G Albihn A Jinnerot T Boqvist S Andersson-Djurfeldt A Sokerya S. Manure management and public health: sanitary and socio-economic aspects among urban livestock-keepers in Cambodia. Sci Total Environ. (2018) 621:193–200. 10.1016/j.scitotenv.2017.11.254 29179075

[40] Martinez J Dabert P Barrington S Burton C Livestock waste treatment systems for environmental quality, food safety, and sustainability. Bioresour Technol. (2009) 100:5527–36. 10.1016/j.biortech.2009.02.038 19369065

[41] Perry B Grace D. The impacts of livestock diseases and their control on growth and development processes that are pro-poor. Philos Trans R Soc Lond B Biol Sci. (2009) 364:2643–55. 10.1098/rstb.2009.0097 19687035 PMC2865091

[42] Rushton J Viscarra R Guerne Bleich E McLeod A Impact of avian influenza outbreaks in the poultry sectors of five South East Asian countries (Cambodia, Indonesia, Lao PDR, Thailand, Viet Nam) outbreak costs, responses and potential long term control. World's Poultry Sci J. (2005) 61:491–514. 10.1079/WPS200570

[43] Rojas-Downing MM Nejadhashemi AP Harrigan T Woznicki SA Climate change and livestock: Impacts, adaptation, and mitigation. Clim Risk Manag. (2017) 16:145–63. 10.1016/j.crm.2017.02.001

[44] FAO The Impact of disasters and crises on agriculture and Food Security. Rome: FAO (2018).

[45] Eckstein D Künzel V Schäfer L Global Climate Risk Index 2018. (2018). Retrieved from: https://germanwatch.org/de/14638 (accessed August 5, 2019).

[46] Ministry of Natural Resources and Environment The Initial Biennial Updated Report of Vietnam to the United Nations Framework Convention on Climate Change. Hanoi: Ministry of Natural Resources and Environment (2014).

[47] FAO FAO's Programme for Gender Equality in Agriculture and Rural development. FAO (2009).

[48] Wilson RT Domestic livestock in African cities: production, problems and prospects. Open Urban Studies Demography J. (2018) 4:1–14. 10.2174/2352631901804010001

